# Relationship between Age and Value of Information for a Noisy Ornstein–Uhlenbeck Process

**DOI:** 10.3390/e23080940

**Published:** 2021-07-23

**Authors:** Zijing Wang, Mihai-Alin Badiu, Justin P. Coon

**Affiliations:** Department of Engineering Science, University of Oxford, Oxford OX1 3PJ, UK; zijing.wang@balliol.ox.ac.uk (Z.W.); mihai.badiu@eng.ox.ac.uk (M.-A.B.)

**Keywords:** value of information, age of information, noisy Ornstein–Uhlenbeck process

## Abstract

The age of information (AoI) has been widely used to quantify the information freshness in real-time status update systems. As the AoI is independent of the inherent property of the source data and the context, we introduce a mutual information-based value of information (VoI) framework for hidden Markov models. In this paper, we investigate the VoI and its relationship to the AoI for a noisy Ornstein–Uhlenbeck (OU) process. We explore the effects of correlation and noise on their relationship, and find logarithmic, exponential and linear dependencies between the two in three different regimes. This gives the formal justification for the selection of non-linear AoI functions previously reported in other works. Moreover, we study the statistical properties of the VoI in the example of a queue model, deriving its distribution functions and moments. The lower and upper bounds of the average VoI are also analysed, which can be used for the design and optimisation of freshness-aware networks. Numerical results are presented and further show that, compared with the traditional linear age and some basic non-linear age functions, the proposed VoI framework is more general and suitable for various contexts.

## 1. Introduction

Nowadays, there are more and more real-time monitoring and control applications, such as industrial control, Internet of Things, autonomous driving and so on. Such applications are modelled as status update systems in which sensors need to continuously monitor a targeted random process, and the sampled status updates are required to be transmitted through the communication network to a remote destination in a timely manner to enable precise control and management. Therefore, the freshness of data has emerged as an important part of network research.

The age of information (AoI) is proposed as a novel end-to-end metric in [1,2] to evaluate the timeliness of status updates from the receiver’s perspective. The AoI is defined as the time difference between the current time and the generation time of the last received status update. The AoI and its variants (e.g., the average AoI and the peak AoI) are widely used as tools to improve the system-level data freshness by optimising the sampling and link scheduling in a variety of emerging networks [3,4,5,6,7,8]. Moreover, there are many works exploring the AoI in the context of different queue systems. General expressions of the average AoI were derived in [1], and the stationary distribution of the AoI was studied in [9,10] for first-come-first-serve (FCFS) M/M/1, M/D/1 and D/M/1 queue disciplines. The statistical characterisation and violation probability of the AoI were treated in [11,12] for last-come-first-serve (LCFS) queue disciplines. The influence of the queue’s buffer size, packet management and service pre-emption on the AoI and its distribution was investigated in [13,14,15].

However, the basic notion of the AoI grows linearly with a unit slope as time goes by, and it is independent of the context and the inherent characterisation of the targeted random process (e.g., the correlation property of the underlying source data). In light of these issues, the concept of the value of information (VoI) has begun to be studied, which emphasises the idea that in some cases, old information may still have value while even fresh information may hold little value, as different sources require different update frequency.

The idea of a non-linear age has become a common approach to evaluate information value [16]. The concept of the “age penalty” was proposed in [17], where it was assumed to be a non-decreasing function of the AoI and provided a general way to measure the dissatisfaction of the staleness of information. Closed-form expressions of the general penalty functions were studied in energy harvesting networks in [18]. In [19,20,21], three specific penalty functions (exponential, linear and logarithmic functions) and their statistical characterisations were further investigated. Moreover, the connection of the AoI with signal processing and information theory has received much attention, as it can provide a theoretical basis for non-linear age functions. The mean square error (MSE) for remote estimation can add non-linearity, and it was used to evaluate the information value in [22,23,24,25]. The relationship between the AoI and the MSE was studied in the Wiener process [22] and the Ornstein–Uhlenbeck (OU) process [23]. It is interesting to note that the age-optimal sampling policy was not equivalent to the MSE-optimal sampling. The mutual information was utilised in [26] to quantify the timeliness of data, and the optimal sampling policy was explored for a Markov source. In [26], the samples were assumed to be directly observable when they were received. In practice, samples at the source can be corrupted by noise, errors or measurements, and thus, they may be latent at the receiver. However, properties of the information value in hidden Markov models have not been explicitly studied. Furthermore, the authors in [20] proposed that age penalty functions can be chosen and adjusted, according to the autocorrelation of the underlying random process, but theoretical interpretation or formal justification for how to choose non-linear functions and how they relate to the correlation of the underlying process were not provided.

In our previous work [27], we proposed a mutual information-based value of information framework for hidden Markov models and started to explore it in the context of a noisy OU process. We obtained the closed-form expression of the VoI, which relates to the correlation of the process under observation at the source and the noise in the transmission environment, but we did not investigate its relationship to the AoI and its statistical characterisations in more depth. In this paper, the connection of the proposed VoI with the AoI is studied for a noisy OU process. The OU process is considered, as it is an important continuous-time, stationary, Markov and Gaussian random process, which is practical to represent many real-world applications [28]. For example, it can be used to model the mobility of a drone that moves towards a target point but experiences positional fluctuations in unmanned aerial vehicle (UAV) networks. In this work, we give the formal justification for how the correlation and the noise in the context affect the VoI and its relationship to AoI, and obtain the functional dependency between them. We show that the proposed VoI framework is a general one that includes the special sample cases given in [20], and it is suitable to be applied in different network settings. Moreover, we study the VoI in a FCFS M/M/1 queue model, deriving the probability density function (PDF), cumulative distribution function (CDF), average VoI and moment-generating function (MGF). We also derive the upper and lower bounds of the average VoI, which are tractable and useful for the design and optimisation of freshness-aware applications. Through all of these results, we provide a clear statistical framework linking the VoI to the AoI and a formal justification for the selection of non-linear age functions.

The rest of this paper is organised as follows. The VoI formalism in the noisy OU process model is introduced in Section 2. Relationships between the VoI and the AoI for different network settings are investigated in Section 3. The statistical characterisation of the VoI in the FCFS M/M/1 queue model is given in Section 4. Numerical results are provided in Section 5. Conclusions are drawn in Section 6.

## 2. VoI with Application to OU Processes

Here, we provide a brief introduction to the VoI framework that is used in this paper, and we recount key results reported in [27] that will be used later in the paper.

### 2.1. VoI Definition

We consider a real-time status update system with a pair of transmitter and receiver nodes. The source samples the data of a targeted random process $\left\{X_{t}\right\}$ and sends status updates to the receiver node for further analysis. Denote $X_{t_{i}}$ as the *i*-th status update of the underlying random process. Denote $Y_{t_{i}^{\prime }}$ as the corresponding observation which is captured in the observed random process $\left\{Y_{t}\right\}$. Here, $t_{i}$ represents the sampling time of the *i*-th sample, and $t_{i}^{\prime }$ represents its receiving time. We consider a latent variable model in which the observation $Y_{t_{i}^{\prime }}$ may be different from the initial value, as the update $X_{t_{i}}$ can be negatively affected by the transmission noise, error or measurement when it is received by the destination in the real world.

In this paper, the notion of the value of information is defined as the mutual information between the current status of the process under observation at the transmitter and a sequence of noisy measurements recorded by the receiver. Specifically, the VoI at the time *t* is given as the following:

(1) $$v\left(t\right)=I\left(X_{t};Y_{t_{n}^{\prime }},\ldots ,Y_{t_{n-m+1}^{\prime }}\right),\;t>t_{n}^{\prime }.$$

Here, *n* is denoted as the index of the last received update during the period (0,t). We look back in time, and the most recent *m* of *n* noisy observations (m≤n) are utilised to evaluate the information value. This definition gives the interpretation of the reduction in the uncertainty of the current hidden status, given that we have some past noisy measurements.

### 2.2. Noisy OU Process Model

We assume the random process $\left\{X_{t}\right\}$ under observation is an Ornstein–Uhlenbeck process, which can be used to represent the mean reversion behaviour in practice. The underlying OU process satisfies the following stochastic differential equation:

(2) $$d\;X_{t}=\kappa \left(\theta -X_{t}\right)d\;t+\sigma d\;W_{t}.$$

Here, κ (κ>0) is the rate of mean reversion, which can be used to represent the correlation property of status updates, θ is the long-term mean, σ is the volatility of the random fluctuation, and $\left\{W_{t}\right\}$ is the Wiener process. We assume that the initial value $X_{0}$ is a Gaussian variable with mean θ and variance $\frac{\sigma^{2}}{2\kappa }$.

We assume this OU process $\left\{X_{t}\right\}$ is observed through an additive noise channel, and the corresponding noisy observation is defined as the following:

(3) $$Y_{t_{i}^{\prime }}=X_{t_{i}}+N_{t_{i}^{\prime }},$$

where $N_{t_{i}^{\prime }}$ is the sample of the noise process taken by the receiver at $t_{i}^{\prime }$. Here, the samples $\left\{N_{t_{i}^{\prime }}\right\}$ are assumed to be independent Gaussian variables with zero mean and constant variance $\sigma_{n}^{2}$. In reality, it can represent the measurement or error that undermines the status update $X_{t_{i}}$ of the underlying OU process.

### 2.3. VoI for the Noisy OU Process

Based on the model we described, the samples of the underlying OU process are jointly Gaussian and the noise samples are also Gaussian variables, which allow us to calculate the VoI in our previous work [27]. The VoI for the noisy OU process is given as follows:

(4) $$v\left(t\right)=-\frac{1}{2}\log\left(1-e^{-2\kappa (t-t_{n})}+e^{-2\kappa (t-t_{n})}\frac{\det(A_{mm})}{\gamma \det(A)}\right),\;t>t_{n}^{\prime }.$$

Here, $A=\sigma_{n}^{2}\Sigma_{X}^{-1}+I$ where $\Sigma_{X}^{-1}$ represents the covariance matrix of the vector $X={\left[X_{t_{n-m+1}},\ldots ,X_{t_{n}}\right]}^{T}$, and I represents the identity matrix of size *m*. $A_{ij}$ represents the (m−1)×(m−1) matrix constructed by deleting the *i*th row and the *j*th column of the matrix A, and γ is denoted as the ratio of the variance of the OU process and the variance of the noise, i.e., the following:

(5) $$\gamma =\frac{\mathrm{Var}[X_{t_{i}}]}{\mathrm{Var}[N_{t_{i}^{\prime }}]}=\frac{\sigma^{2}}{2\kappa \sigma_{n}^{2}}.$$

The parameter γ is similar to the concept of the signal-to-noise ratio (SNR) in a communication system. In the following, the concept “SNR” refers to this parameter, which is used to compare the randomness in the OU process and the noise in the communication channel.

## 3. Relationship between VoI and AoI

The result given in (4) shows the general expression of the VoI in the noisy OU process. In this section, we consider a special case with a single observation (m=1) and explore the relationship between the proposed VoI and the AoI. In the definition of the AoI, we consider that the time instant $t_{n}$ is fixed, i.e., we view the AoI as deterministic. What we do here is to create a relationship between the VoI and the conditional AoI (i.e., the AoI conditioned on the most recent sample time).

The concept of the AoI is given as follows [1]:

(6) $$A\left(t\right)=t-t_{n},\;t>t_{n}^{\prime }.$$

In the noisy OU process, when m=1, the VoI in (4) can be simplified as follows:

(7) $$v\left(t\right)=-\frac{1}{2}\log\left(1-\frac{\gamma }{1+\gamma }e^{-2\kappa (t-t_{n})}\right),\;t>t_{n}^{\prime },$$

which is supported by the following:

(8) $$0\leq v\leq \frac{1}{2}\log\left(1+\gamma \right).$$

Therefore, the VoI is further written as a function of the AoI. Let a=A(t); then, the VoI can be written as follows:

(9) $$V\left(a\right)=-\frac{1}{2}\log\left(1-\frac{\gamma }{1+\gamma }e^{-2\kappa a}\right).$$

The VoI in (9) and its relationship to the AoI can be largely affected by system parameters. Fixing the random fluctuation parameter $\sigma^{2}$ of the OU process, the SNR γ relates to two parameters, κ and $\sigma_{n}^{2}$. κ can be used to represent the correlation property of the underlying OU process. If κ is small, the status updates are highly correlated; as κ increases, they become less correlated. $\sigma_{n}^{2}$ represents the noise level in the transmission environment. If $\sigma_{n}^{2}$ is small, the underlying hidden Markov process is dominant, and the VoI approaches its Markov counterpart in the OU model; otherwise, the noise process is dominant. In the following part, the relationship between the VoI and AoI in different SNR regimes is investigated, and we have the following corollaries.

**Corollary** **1.** *In the low SNR regime, the VoI can be approximated as an exponential function of the AoI, which is given by the following:*

(10) $$V\left(a\right)\approx \frac{\gamma }{2(1+\gamma )}e^{-2\kappa a}.$$

**Proof.**  In the low SNR regime (small γ), large κ and $\sigma_{n}^{2}>0$ (or large $\sigma_{n}^{2}$ and κ>0) can lead to small SNR in (5). When γ approaches 0, the term $\frac{\gamma }{1+\gamma }e^{-2\kappa a}$ in (9) is small. For small *x*, we have log(1+x)≈x, thus the result in (10) is obtained. ☐

In the low SNR regime, the dependency between the VoI and AoI is exponential. Less correlated samples or large noise can negatively affect the VoI at the receiver, thus the approximated VoI decreases faster as the AoI increases. For a less correlated data source, even fresh updates may contain little valuable information about the underlying OU process. For a high level of noise, status updates are corrupted, due to the indirect observation.

**Corollary** **2.** *In the high SNR regime resulting from high correlation, the VoI can be approximated as a logarithmic function of the AoI, which is given by the following:*

(11) $$V\left(a\right)\approx -\frac{1}{2}\log\left(2\kappa \gamma a+1\right)+\frac{1}{2}\log\left(1+\gamma \right).$$

**Proof.**  For small *x*, we have $e^{x}\approx 1+x$. Therefore, when κ→0 in (9), $e^{-2\kappa a}\approx 1-2\kappa a$. ☐

For highly correlated status updates, the VoI is expressed as a logarithmic function, and this means that the VoI decreases slower as the AoI increases. In this case, correlated updates can be transmitted under good channel conditions, thus old samples may still hold enough valuable information.

**Corollary** **3.** *In the intermediate SNR regime where κ→0, $\sigma_{n}^{2}\to \infty$ with $\kappa \sigma_{n}^{2}$ being constant, the VoI can be approximated as a linear function of the AoI, which is given by the following:*

(12) $$V\left(a\right)\approx -\kappa \gamma a+\frac{1}{2}\log\left(1+\gamma \right).$$

*In the intermediate SNR regime where κ→∞, $\sigma_{n}^{2}\to 0$ with $\kappa \sigma_{n}^{2}$ being constant, the VoI can be approximated as an exponential function of the AoI, which is given by the following:*

(13) $$V\left(a\right)\approx \frac{\gamma }{2(1+\gamma )}e^{-2\kappa a}.$$

**Proof.**  The result in (12) can be derived from Corollary 2 directly. When $\sigma_{n}^{2}\to \infty$, the term 2κγa in (11) is small. Therefore, we have log(2κγa+1)≈2κγa. The result in (13) matches Corollary 1. When κ→∞, the term $e^{-2\kappa a}$ in (9) is small. For small *x*, we have log(1+x)≈x, thus the result in (13) is obtained. ☐

The three corollaries stated above provide the compelling insight into the adoption of non-linear AoI functions. In some existing works, exponential and logarithmic non-linear age functions are widely utilised to measure the information value, but they do not give the formal justification for why these functions are selected. Corollaries 1 to 3 provide a theoretic interpretation and explain how the correlation, noise and SNR affect the VoI and its relationship to the AoI in the noisy OU process. Generally, low SNR and high SNR conditions yield exponential and logarithmic relationships. The intermediate SNR regime yields an exponential or linear relationship, which depends on the value of noise and correlation. Therefore, the proposed VoI framework is more complete, general and appropriate to measure the timeliness of information in different SNR regimes.

## 4. Statistical Properties of the VoI in the M/M/1 Queue Model

Equations (10)–(13) show general relationships between the VoI and the AoI in the noisy OU process. In this section, we relax the “fixed time instants” restriction given in Section 3 and view the AoI as a random variable to study the distribution of the VoI. We explore the VoI in a specific FCFS M/M/1 queue system and derive its statistical properties (including the PDF, CDF, expectation value and MGF).

### 4.1. Distribution of the VoI

We assume that status updates of the underlying OU process are transmitted through a FCFS M/M/1 queue in which they are sampled as a rate λ Poisson process, and the service time is a rate μ exponential process (λ<μ). Let random variables $S_{i}=t_{i}-t_{i-1}$ (2≤i≤n) be the sampling interval of two packets, which are independent and identically distributed (i.i.d.) exponential random variables with $E\left[S\right]=\frac{1}{\lambda }$. Similarly, service times of status updates are also i.i.d. exponential random variables with mean $\frac{1}{\mu }$. In the example of the M/M/1 queue, the stationary distribution of the AoI was studied in [11] and the PDF and CDF of the AoI are given as follows:

(14) $$f_{A}\left(a\right)=\mu \left[\frac{\mu -\lambda }{\mu }e^{-(\mu -\lambda )a}-\left(\frac{\mu }{\mu -\lambda }+\lambda a-\frac{\lambda }{\mu }\right)e^{-\mu a}+\frac{\lambda }{\mu -\lambda }e^{-\lambda a}\right],$$

(15) $$F_{A}\left(a\right)=1-e^{-(\mu -\lambda )a}+\left(\frac{\mu }{\mu -\lambda }+\lambda a\right)e^{-\mu a}-\frac{\mu }{\mu -\lambda }e^{-\lambda a}.$$

It can be seen that the distribution of the AoI only relates to the queue discipline, which means that it is independent of the inherent statistical characterisations of the underlying random process. As for the distribution of the VoI of a latent OU process with a single observation, we can state the following propositions.

**Proposition** **1.** *In the M/M/1 queue model, the PDF of the VoI for the noisy OU process is given by the following:*

(16) $$\begin{array}{c} f_{V}\left(v\right)=\frac{\mu e^{-2v}}{\kappa (1-e^{-2v})}[\frac{\mu -\lambda }{\mu }r{\left(v\right)}^{\frac{\mu -\lambda }{2\kappa }}-\left(\frac{\mu }{\mu -\lambda }-\frac{\lambda }{\mu }-\frac{\lambda }{2\kappa }\log r\left(v\right)\right)r{\left(v\right)}^{\frac{\mu }{2\kappa }}\\ +\frac{\lambda }{\mu -\lambda }r{\left(v\right)}^{\frac{\lambda }{2\kappa }}], \end{array}$$

*where r(v) is denoted as follows:*

(17) $$r\left(v\right)=\frac{\left(1+\gamma \right)\left(1-e^{-2v}\right)}{\gamma }.$$

**Proof.**  Since (9) is a monotonically decreasing function, the PDF of the VoI can be calculated by the following:

(18) $$f_{V}\left(v\right)=f_{A}\left(V^{-1}\left(v\right)\right)\left|\frac{d}{d\;v}\left(V^{-1}\left(v\right)\right)\right|.$$

Here, $V^{-1}$ denotes the inverse function of the VoI given in (9), which can be written as follows:

(19) $$V^{-1}\left(v\right)=-\frac{1}{2\kappa }\log\left(\frac{\left(1+\gamma \right)\left(1-e^{-2v}\right)}{\gamma }\right),$$

and we have the following:

(20) $$\frac{d}{d\;v}\left(V^{-1}\left(v\right)\right)=-\frac{e^{-2v}}{\kappa (1-e^{-2v})}.$$

Therefore, the PDF of the VoI given in (16) is obtained by substituting (19), (14) and (20) into (18). ☐

**Proposition** **2.** *In the M/M/1 queue model, the CDF of the VoI for the noisy OU process is given as follows:*

(21) $$F_{V}\left(v\right)=r{\left(v\right)}^{\frac{\mu -\lambda }{2\kappa }}-\left(\frac{\mu }{\mu -\lambda }-\frac{\lambda }{2\kappa }\log r\left(v\right)\right)r{\left(v\right)}^{\frac{\mu }{2\kappa }}+\frac{\mu }{\mu -\lambda }r{\left(v\right)}^{\frac{\lambda }{2\kappa }}.$$

**Proof.**  The CDF is obtained directly by the integral of the PDF, i.e., $F_{V}\left(v\right)=P\left(V\leq v\right)=\int_{0}^{v}f_{V}\left(x\right)d\;x$. ☐

Propositions 1 and 2 show that the distribution of the VoI relates to the sampling rate λ, service rate μ, correlation parameter κ and noise parameter $\sigma_{n}^{2}$, while the AoI distribution only relates to parameters λ and μ for the M/M/1 queue system.

The CDF of the VoI given in Proposition 2 can be interpreted as the “VoI outage probability”, i.e., the probability that the VoI is smaller than a given threshold. It is interesting to note that Proposition 2 implies that the VoI outage probability is a monotonically decreasing function of the service rate μ, and it converges to $r{\left(v\right)}^{\frac{\lambda }{2\kappa }}$ as μ goes to infinity. The proof of this is given in Appendix A.1. The reason for this decreasing nature of the VoI with μ is predictable because one would expect the information value to increase if the service time in the queue reduces.

Proposition 2 also implies that the VoI outage probability first decreases and then increases as the sampling rate λ increases. The optimal sampling rate $\lambda^{*}$ satisfies $\frac{\partial P(V\leq v)}{\partial \lambda }{|}_{\lambda =\lambda^{*}}=0$. The proof of this is provided in Appendix A.2. It is not surprising that small sampling rate λ can lead to high outage, due to the lack of fresh updates at the source. It is interesting to find that large sampling rate can also lead to high outage probability, due to the traffic congestion in the queue.

### 4.2. Moments and Bounds

In this subsection, we derive the expectation and two bounds of the VoI with a single observation, and calculate the moment-generating function of the VoI. We can state the following two propositions.

**Proposition** **3.** *In the M/M/1 queue model, the average VoI for the noisy OU process is given as the following:*

(22) $$\begin{array}{c} E\left[V\right]=\frac{1}{2}[\log\left(1+\gamma \right)-g_{1}\left(\frac{\gamma }{1+\gamma },\frac{\mu -\lambda }{2\kappa }\right)-\frac{\mu }{\mu -\lambda }g_{1}\left(\frac{\gamma }{1+\gamma },\frac{\lambda }{2\kappa }\right)\\ +\left(\frac{\mu }{\mu -\lambda }+\frac{\lambda }{2\kappa }\log\frac{\gamma }{1+\gamma }\right)g_{1}\left(\frac{\gamma }{1+\gamma },\frac{\mu }{2\kappa }\right)-\frac{\lambda }{2\kappa }g_{2}\left(\frac{\gamma }{1+\gamma },\frac{\mu }{2\kappa }\right)], \end{array}$$

*where two functions $g_{1}\left(x,y\right)$ and $g_{2}\left(x,y\right)$ are defined for x>0 and y>0 with the following:*

(23) $$g_{1}\left(x,y\right)=\frac{1}{x^{y}}\int_{0}^{x}\frac{z^{y}}{1-z}d\;z,$$

(24) $$g_{2}\left(x,y\right)=\frac{1}{x^{y}}\int_{0}^{x}\frac{z^{y}\log z}{1-z}d\;z.$$

*Moreover, the average VoI is lower bounded by the following:*

(25) $$E\left[V\right]\geq -\frac{1}{2}\log\left[1-\frac{\gamma }{1+\gamma }\left(\frac{\frac{\mu -\lambda }{2\kappa }}{\frac{\mu -\lambda }{2\kappa }+1}-\frac{\frac{\mu -\lambda }{2\kappa }\left(\frac{\mu +\lambda }{2\kappa }+1\right)}{{\left(\frac{\mu }{2\kappa }+1\right)}^{2}\left(\frac{\lambda }{2\kappa }+1\right)}\right)\right],$$

*and it is upper bounded by the following:*

(26) $$\begin{array}{c} E\left[V\right]\leq \frac{1}{2}\left[H\left(\frac{\mu -\lambda }{2\kappa }\right)+\frac{\mu }{\mu -\lambda }H\left(\frac{\lambda }{2\kappa }\right)-\frac{\mu }{\mu -\lambda }H\left(\frac{\mu }{2\kappa }\right)+\frac{\lambda }{2\kappa }\psi^{\left(1\right)}\left(1+\frac{\mu }{2\kappa }\right)\right]. \end{array}$$

Here, H(·) represents the harmonic number and the integral representation is given by the following: $H\left(x\right)=\int_{0}^{1}\frac{1-z^{x}}{1-z}d\;z$ [29]. $\psi^{\left(1\right)}\left(\cdot \right)$ represents the first order polygamma function which is given by $\psi^{\left(1\right)}\left(x\right)=-\int_{0}^{1}\frac{z^{x-1}\log z}{1-z}d\;z$ [30].

**Proof.**  See Appendix B. ☐

This proposition gives two bounds of the average VoI in the noisy OU process. Compared with the general average VoI, the bounds are more tractable and may be useful for network design and optimisation. The details of the bounds are given in Appendix B as stated above.

The lower bound is based on Jensen’s inequality. The equality holds if the VoI is a linear function on the Laplace transform of the AoI ($E[e^{-2\kappa a}]$). In Corollary 1, we show that the dependence between the VoI and $E[e^{-2\kappa a}]$ is approximately linear under the low SNR condition. Therefore, the average VoI approaches this lower bound in the low SNR regime. Moreover, as stated in Appendix B, the upper bound is based on the average VoI in the Markov model. Hence, in the high SNR regime, the upper bound is tight.

**Proposition** **4.** *In the M/M/1 queue, the MGF of the VoI for the noisy OU process is given as follows:*

(27) $$\begin{array}{c} M_{v}\left(t\right)={}_{2}F{}_{1}\left(\frac{\mu -\lambda }{2\kappa },\frac{t}{2};\frac{\mu -\lambda }{2\kappa }+1;\frac{\gamma }{1+\gamma }\right)+\frac{\mu }{\mu -\lambda }{}_{2}F{}_{1}\left(\frac{\lambda }{2\kappa },\frac{t}{2};\frac{\lambda }{2\kappa }+1;\frac{\gamma }{1+\gamma }\right)\\ -\left(\frac{\mu }{\mu -\lambda }-\frac{\lambda }{\mu }\right){}_{2}F{}_{1}\left(\frac{\mu }{2\kappa },\frac{t}{2};\frac{\mu }{2\kappa }+1;\frac{\gamma }{1+\gamma }\right)-\frac{\lambda }{\mu }{}_{3}F{}_{2}\left(\frac{\mu }{2\kappa },\frac{\mu }{2\kappa },\frac{t}{2};\frac{\mu }{2\kappa }+1,\frac{\mu }{2\kappa }+1;\frac{\gamma }{1+\gamma }\right). \end{array}$$

Here, ${}_{p}F_{q}\left(a_{1},\ldots ,a_{p};b_{1},\ldots ,b_{q};z\right)$ represents the generalised hypergeometric function which is given by the following series:

(28) $${}_{p}F_{q}\left(a_{1},\ldots ,a_{p};b_{1},\ldots ,b_{q};z\right)=\sum_{n=0}^{\infty }\frac{{\left(a_{1}\right)}_{n}\ldots {\left(a_{p}\right)}_{n}}{{\left(b_{1}\right)}_{n}\ldots {\left(b_{q}\right)}_{n}}\frac{z^{n}}{n!}.$$

where ${\left(\cdot \right)}_{n}$ represents the Pochhammer symbol, which is given as follows:

(29) $${\left(x\right)}_{n}=\left\{\begin{array}{cc} 1 & n=0\\ \prod_{i=0}^{n-1}\left(x-i\right) & n\geq 1 \end{array}.\right.$$

**Proof.**  See Appendix C. ☐

Moments of the VoI can be obtained by derivatives of the MGF at t=0. The average VoI given in Proposition 3 is the first-order moment and can be derived from the MGF directly. Using this MGF, higher order moments can also be used for the system design and optimisation instead of just utilising the average value.

## 5. Numerical Results

In this section, the relationship between the VoI and AoI and the distribution of the VoI are investigated through Monte Carlo simulations. In the simulation, the volatility parameter $\sigma^{2}$ of the OU model is fixed and set as 1. The sampling times $\left\{t_{i}\right\}$ are randomly generated by the rate λ Poisson process. The service times of each sample are randomly generated by the rate μ exponential process. We set time t=100. For each running round, we record the sampling time of the most recent received update as $t_{n}$, and the AoI and the VoI are calculated by (6) and (7), respectively.

Figure 1, Figure 2 and Figure 3 show the non-linear relationships between the VoI and the AoI under low, high and intermediate SNR conditions, respectively. Figure 4, Figure 5, Figure 6 and Figure 7 illustrate the distribution of the VoI, including the PDF, CDF and the outage probability. Figure 8, Figure 9, Figure 10 and Figure 11 provide the numerical results about the VoI expectation and bounds.

**Figure 1:**
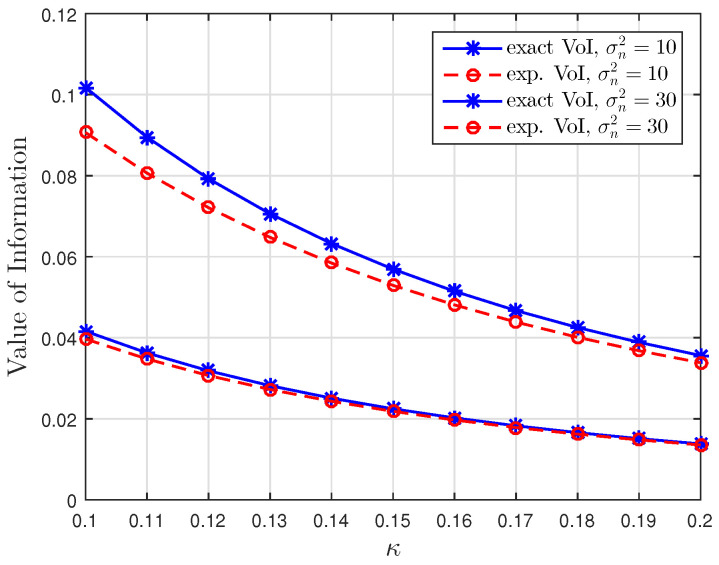
Low SNR regime: Comparison of the exact VoI and the exponential VoI versus κ for $\sigma_{n}^{2}\in \left\{10,30\right\}$ at t=100, sampling rate λ=0.5 and service rate μ=1.

**Figure 2:**
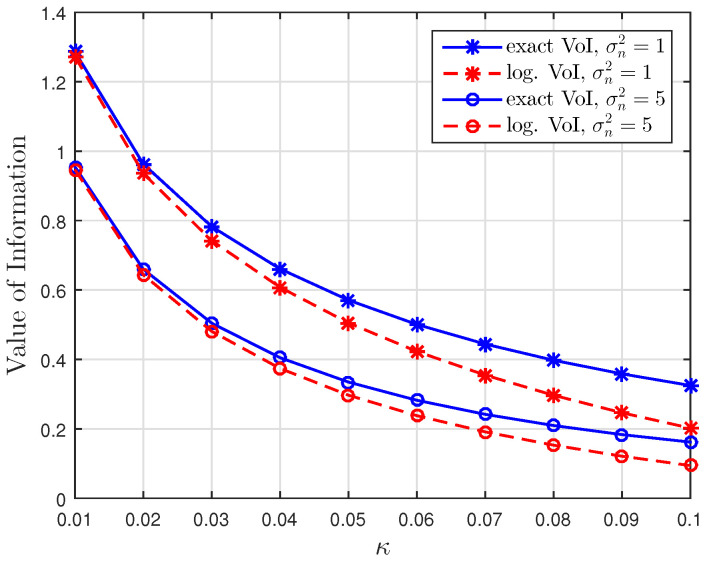
High SNR regime: Comparison of the exact VoI and the logarithmic VoI versus κ for $\sigma_{n}^{2}\in \left\{1,5\right\}$ at t=100, sampling rate λ=0.5 and service rate μ=1.

**Figure 3:**
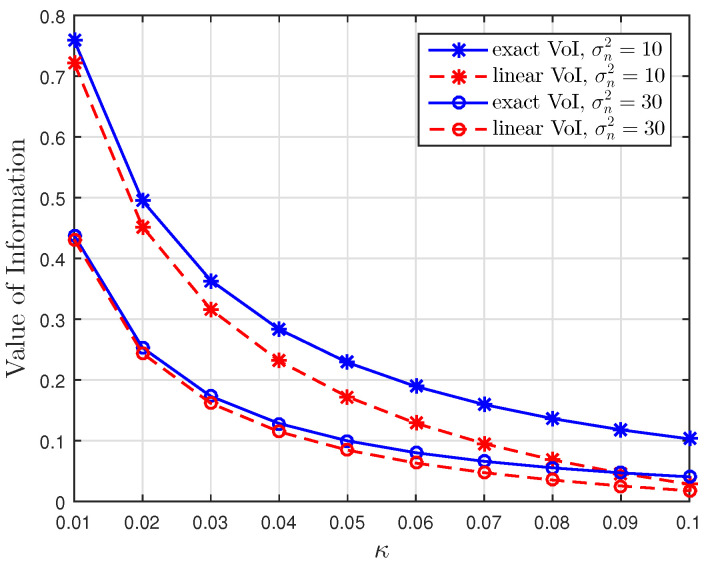
Intermediate SNR regime: Comparison of the exact VoI and the linear VoI versus κ for $\sigma_{n}^{2}\in \left\{10,30\right\}$ at t=100, sampling rate λ=0.5 and service rate μ=1.

Figure 1, Figure 2 and Figure 3 compare the exact VoI in (7) and the approximated VoI for different SNR regimes which are given in (10) to (12). Figure 1 shows that the exponential approximation is suitable when updates are less correlated and the noise is large. Figure 2 shows the opposite behaviour. The logarithmic approximation is more accurate when κ and $\sigma_{n}^{2}$ are small. Figure 3 shows that the linear approximation is accurate when κ is small but $\sigma_{n}^{2}$ is large. These results verify the functional dependencies between VoI and the AoI, which are discussed in Corollaries 1–3, illustrating that the low, high and intermediate SNR conditions yield exponential, logarithmic and linear relationships.

**Figure 4:**
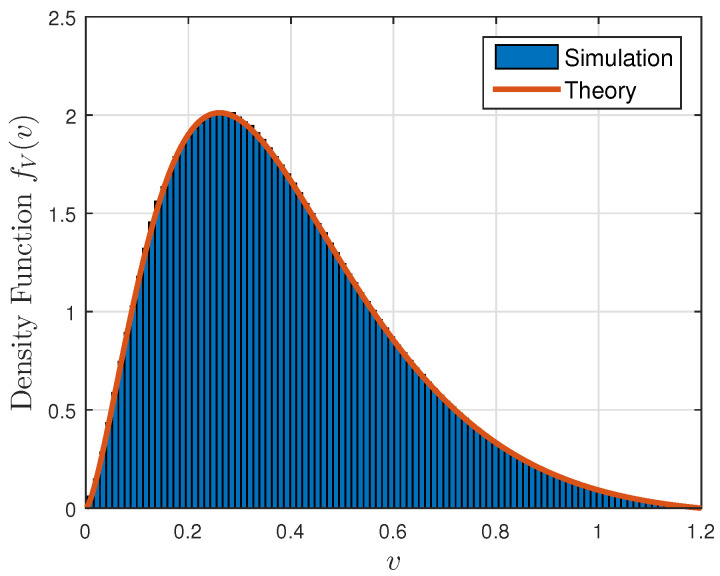
The density function of the VoI; correlation parameter κ=0.1, noise parameter $\sigma_{n}^{2}=0.5$, sampling rate λ=0.5 and service rate μ=1.

**Figure 5:**
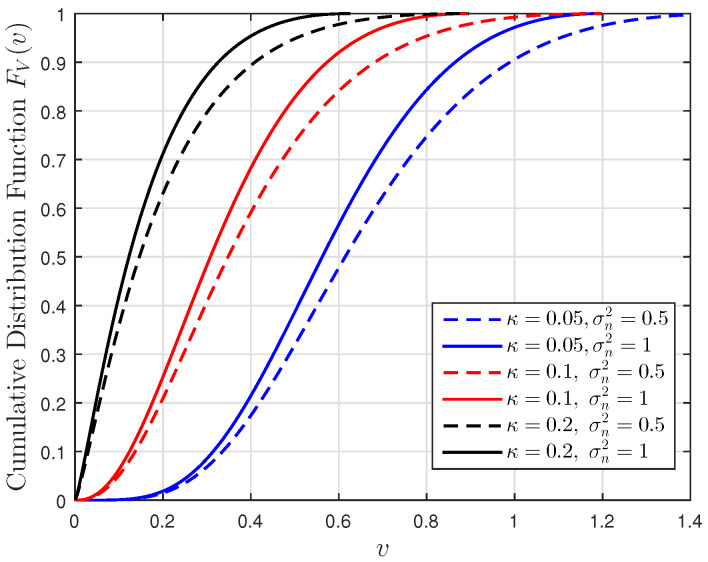
The cumulative distribution function of the VoI versus *v* for $\sigma_{n}^{2}\in \left\{0.5,1\right\}$ and κ∈{0.05,0.1,0.2}; sampling rate λ=0.5 and service rate μ=1.

**Figure 6:**
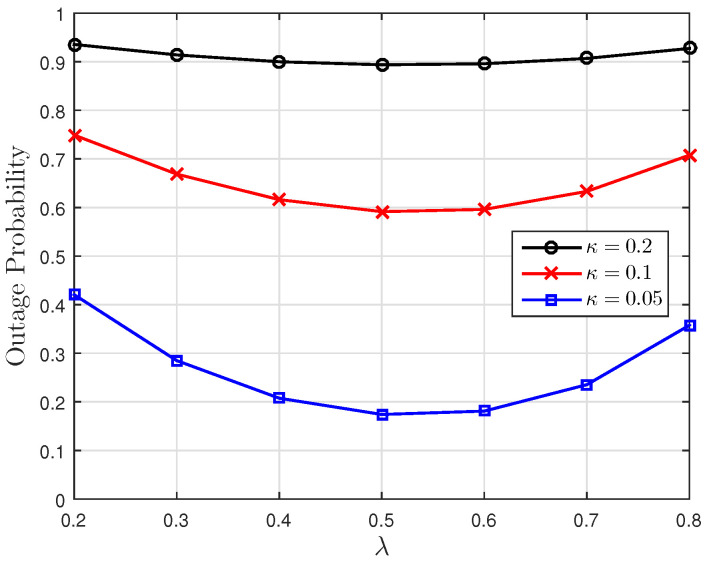
The VoI outage probability versus λ for κ∈{0.05,0.1,0.2}; threshold v=0.4, noise parameter $\sigma_{n}^{2}=0.5$ and service rate μ=1.

**Figure 7:**
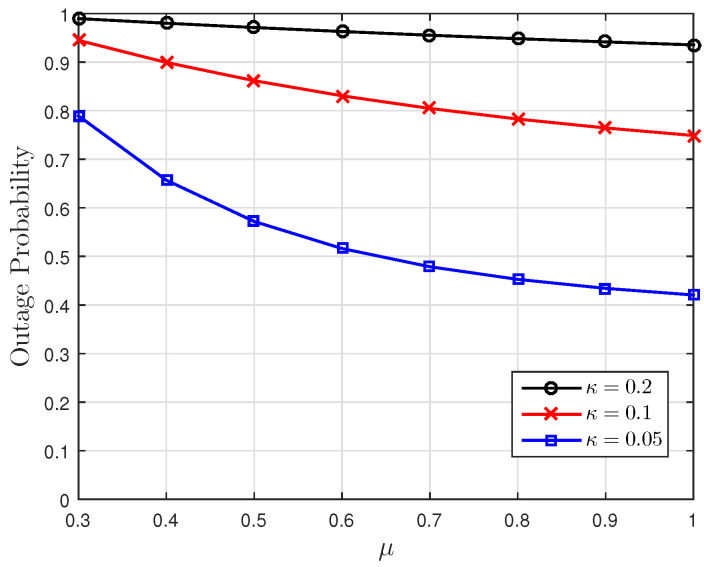
The VoI outage probability versus μ for κ∈{0.05,0.1,0.2}; threshold v=0.4, noise parameter $\sigma_{n}^{2}=0.5$ and sampling rate λ=0.2.

Figure 4 gives the numerical validation of the theoretical PDF given in Proposition 1 and the density of the discrete path of the VoI obtained from the Monte Carlo simulations. Figure 5, Figure 6 and Figure 7 show the VoI outage probability given in Proposition 2 for different system parameters. In Figure 5, the VoI outage probability is high when the status updates are less correlated or when the system experiences large noise. For a particular threshold *v*, Figure 6 shows that either a too-small or too-large sampling rate can lead to a large VoI outage probability. Fixing μ, small λ means that we do not have sufficient newly generated status updates about the underlying OU process for prediction. Large λ means that enough newly generated updates have been sampled at the source, but they have to wait for a longer time, due to the packet congestion in the FCFS queue. Figure 7 shows that VoI outage probability decreases as the service rate μ increases. In the M/M/1 model, λ is smaller than μ. Fixing λ, large μ means that status updates can be served and transmitted more quickly, thus the receiver can hold more valuable information about the underlying process. These two figures verify the discussion given in Proposition 2.

**Figure 8:**
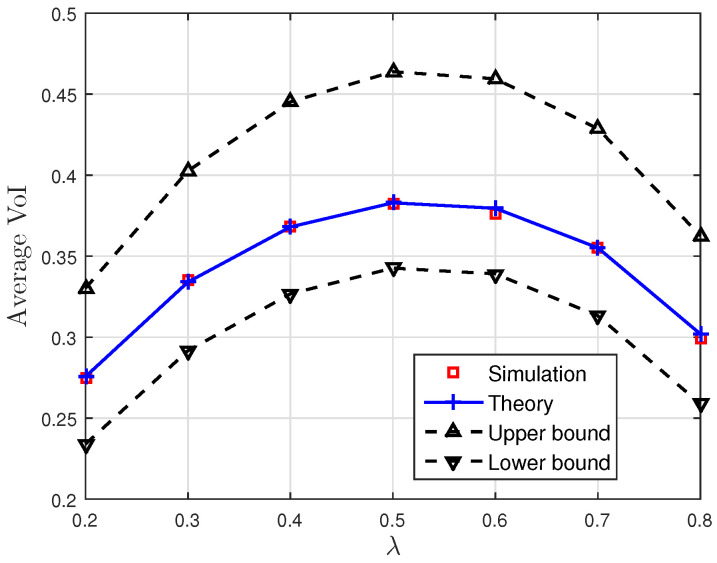
The average VoI and its bounds versus the sampling rate λ; correlation parameter κ=0.1, noise parameter $\sigma_{n}^{2}=0.5$ and service rate μ=1.

**Figure 9:**
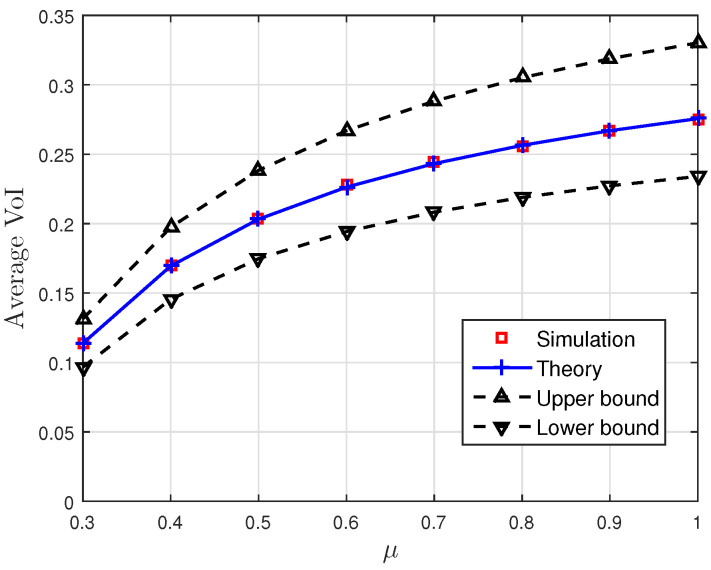
The average VoI and its bounds versus the service rate μ; correlation parameter κ=0.1, noise parameter $\sigma_{n}^{2}=0.5$ and sampling rate λ=0.2.

Figure 8 and Figure 9 show the effect of the sampling rate and service rate on the average VoI and its bounds given in Proposition 3. The average VoI and its bounds first increase and then decrease as λ increases, and they increase as μ increases. This behaviour is similar to the VoI outage and can be explained similar to Figure 6 and Figure 7. Moreover, it can be seen that the theoretical average VoI is consistent with the result obtained from the Monte Carlo simulations.

**Figure 10:**
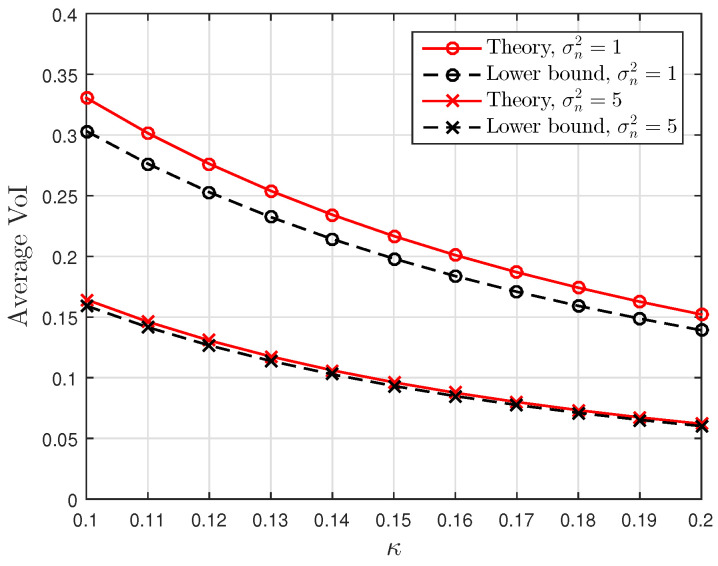
The average VoI and the lower bound versus κ for $\sigma_{n}^{2}\in \left\{1,5\right\}$; sampling rate λ=0.5 and service rate μ=1.

**Figure 11:**
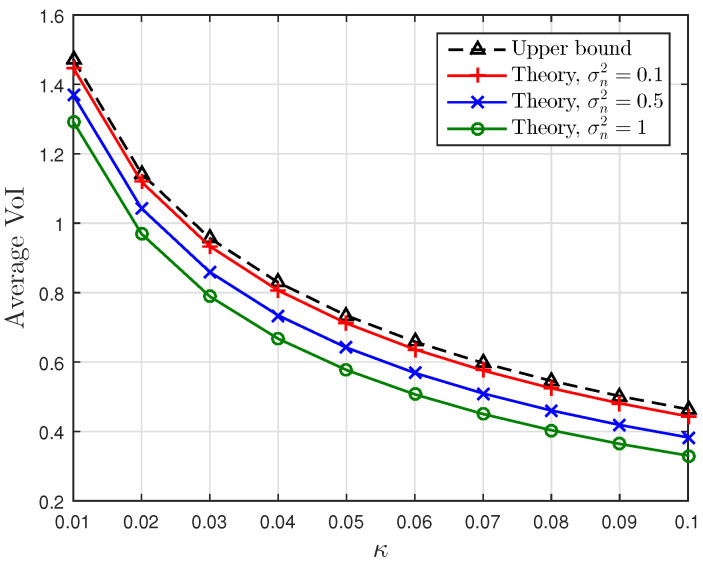
The average VoI and the upper bound versus κ for $\sigma_{n}^{2}\in \left\{0.1,0.5,1\right\}$; sampling rate λ=0.5 and service rate μ=1.

Figure 10 and Figure 11 plot the theoretical average VoI in (22) and the lower and upper bounds in (25) and (26) for different κ and $\sigma_{n}^{2}$. Small noise $\sigma_{n}^{2}$ and small κ can lead to large average VoI. In Figure 10, the gap between the exact value and the lower bound is small for large $\sigma_{n}^{2}$, and it decreases as κ increases. The gap between the exact value and the upper bound in Figure 11 shows the opposite behaviour; the gap narrows as $\sigma_{n}^{2}$ decreases. These two figures verify the discussion given in Proposition 3, illustrating that the average VoI approaches lower and upper bounds in low and high SNR regimes, respectively.

## 6. Conclusions

In this paper, we investigated the dependency between the proposed VoI and the AoI in a noisy OU process. The VoI is defined as the mutual information between the current status of the underlying random process and noisy observations captured by the receiver. Functional relationships between the VoI and the AoI were obtained in low, intermediate and high SNR regimes. Moreover, the distribution and moments of the VoI were investigated in the example of the M/M/1 queue model. Finally, we performed Monte Carlo simulations to obtain numerical validation of the theoretical analysis. The results presented in this paper provide insight into how the correlation and noise in a latent OU process influence the VoI of the observations of that process. We also elucidated the relationship between the VoI and the AoI. Our work has given a mathematical justification for selecting certain non-linear age functions. Future work can be focused on exploring the effect of multiple observations on the VoI and AoI relationship and on estimating the value of the status of the underlying process with multiple observations.

## Data Availability

Not applicable.

## Appendix A

### Appendix A.1. Proof of Monotonicity in μ

First, we prove the monotonicity in μ. For any particular VoI threshold *v*, the derivative of the VoI outage is given as follows:

(A1) $$\begin{array}{c} \frac{\partial P(V\leq v)}{\partial \mu }=\frac{\log r(v)}{2\kappa }\left[r{\left(v\right)}^{\frac{\mu -\lambda }{2\kappa }}-\left(\frac{\mu }{\mu -\lambda }-\frac{\lambda }{2\kappa }\log r\left(v\right)\right)r{\left(v\right)}^{\frac{\mu }{2\kappa }}\right]\\ +\frac{\lambda }{{\left(\mu -\lambda \right)}^{2}}\left(r{\left(v\right)}^{\frac{\mu }{2\kappa }}-r{\left(v\right)}^{\frac{\lambda }{2\kappa }}\right). \end{array}$$

For simplicity, let $x_{1}=\frac{\lambda }{2\kappa }\log r\left(v\right)$ and $x_{2}=\frac{\mu }{2\kappa }\log r\left(v\right)$. Then, (A1) can be written as follows:

(A2) $$\begin{array}{c} \frac{\partial P(V\leq v)}{\partial \mu }=\frac{\log r(v)}{2\kappa }\left[e^{x_{2}-x_{1}}+\left(x_{1}-\frac{x_{2}}{x_{2}-x_{1}}\right)e^{x_{2}}\right]+\frac{\log r(v)}{2\kappa }\frac{x_{1}}{{\left(x_{2}-x_{1}\right)}^{2}}\left(e^{x_{2}}-e^{x_{1}}\right)\\ =\frac{\log r(v)}{2\kappa }e^{x_{2}}\left[e^{-x_{1}}+x_{1}-\frac{x_{2}}{x_{2}-x_{1}}+\frac{x_{1}\left(1-e^{x_{1}-x_{2}}\right)}{{\left(x_{2}-x_{1}\right)}^{2}}\right]. \end{array}$$

Since λ<μ and 0<r(v)<1, thus $x_{2}<x_{1}<0$ and logr(v)<0. Moreover, for any *x*, we have $e^{x}\geq 1+x$. Therefore, (A2) can be further given as follows:

(A3) $$\begin{array}{cc} \frac{\partial P(V\leq v)}{\partial \mu } & \leq \frac{\log r(v)}{2\kappa }e^{x_{2}}\left[1-\frac{x_{2}}{x_{2}-x_{1}}+\frac{x_{1}\left(x_{2}-x_{1}\right)}{{\left(x_{2}-x_{1}\right)}^{2}}\right]\\ \; & =0. \end{array}$$

As the derivative is non-positive, the VoI outage is a monotonic function of μ.

### Appendix A.2. Proof of Optimal λ Exists

Next, we prove that the optimal sampling rate exists. The derivative of the VoI outage with respect to λ is given as follows:

(A4) $$\begin{array}{c} \frac{\partial P(V\leq v)}{\partial \lambda }=-\frac{\log r(v)}{2\kappa }\left(r{\left(v\right)}^{\frac{\mu -\lambda }{2\kappa }}-r{\left(v\right)}^{\frac{\mu }{2\kappa }}-\frac{\mu }{\mu -\lambda }r{\left(v\right)}^{\frac{\lambda }{2\kappa }}\right)\\ \;\;\;-\frac{\mu }{{\left(\mu -\lambda \right)}^{2}}\left(r{\left(v\right)}^{\frac{\mu }{2\kappa }}-r{\left(v\right)}^{\frac{\lambda }{2\kappa }}\right)\\ \;\;\;\;\;\;\;\;\;\;=-\frac{\log r(v)}{2\kappa }\left[e^{x_{2}-x_{1}}-e^{x_{2}}-\frac{x_{2}e^{x_{1}}}{x_{2}-x_{1}}+\frac{x_{2}\left(e^{x_{2}}-e^{x_{1}}\right)}{{\left(x_{2}-x_{1}\right)}^{2}}\right]. \end{array}$$

When λ approaches 0, we can write the following:

(A5) $$\lim_{x_{1}\to 0}\frac{\partial P(V\leq v)}{\partial \lambda }=-\frac{\log r(v)}{2\kappa }\left(\frac{e^{x_{2}}-1}{x_{2}}-1\right)\leq 0.$$

When λ approaches μ, we have the following:

(A6) $$\lim_{x_{1}\to x_{2}}\frac{\partial P(V\leq v)}{\partial \lambda }=-\frac{\log r(v)}{2\kappa }\left[1-e^{x_{2}}\left(1-\frac{x_{2}}{2}\right)\right]\geq -\frac{\log r(v)}{2\kappa }\left(1-e^{\frac{x_{2}}{2}}\right)\geq 0.$$

We show that the VoI outage probability decreases with λ when λ is small, and increases when λ is large. Therefore, there exists the optimal sampling rate $\lambda^{*}$, and the minimum outage probability is achieved when the derivative in (A4) is 0.

## Appendix B. Proof of Proposition 3

The average VoI can be obtained directly by the following:

(A7) $$\begin{array}{c} E\left[V\right]=\int_{0}^{\frac{1}{2}\log\left(1+\gamma \right)}vf_{V}\left(v\right)d\;v=-\frac{\mu }{4\kappa }\int_{0}^{1}\log\left(1-\frac{\gamma }{1+\gamma }r\right)[\frac{\mu -\lambda }{\mu }r^{\frac{\mu -\lambda }{2\kappa }-1}\\ +\frac{\lambda }{\mu -\lambda }r^{\frac{\lambda }{2\kappa }-1}-\left(\frac{\mu }{\mu -\lambda }-\frac{\lambda }{\mu }-\frac{\lambda }{2\kappa }\log r\right)r^{\frac{\mu }{2\kappa }-1}]d\;r. \end{array}$$

Here, for the given *x* and *y*, we have the following:

(A8) $$\begin{array}{cc} \int_{0}^{1}\log\left(1-xr\right)\cdot r^{y-1}d\;r & =\frac{1}{x^{y}}\int_{0}^{x}\log\left(1-z\right)\cdot z^{y-1}d\;z\\ \; & =\frac{1}{x^{y}}\frac{z^{y}}{y}\log\left(1-z\right){|}_{z=0}^{z=x}+\frac{1}{yx^{y}}\int_{0}^{x}\frac{z^{y}}{1-z}d\;z\\ \; & =\frac{1}{y}\left[\log\left(1-x\right)+g_{1}\left(x,y\right)\right], \end{array}$$

and

(A9) $$\begin{array}{c} \int_{0}^{1}\log r\log\left(1-xr\right)\cdot r^{y-1}d\;r\\ =\frac{1}{x^{y}}\left(\int_{0}^{x}\log z\cdot \log\left(1-z\right)\cdot z^{y-1}d\;z-\log x\int_{0}^{x}\log\left(1-z\right)\cdot z^{y-1}d\;z\right)\\ =\frac{1}{x^{y}}\frac{z^{y}}{y}\log\left(1-z\right)\cdot \log z{|}_{z=0}^{z=x}-\frac{\log x}{x^{y}}\int_{0}^{x}\log\left(1-z\right)\cdot z^{y-1}dd\;z\\ -\frac{1}{yx^{y}}\int_{0}^{x}z^{y}\left(\frac{\log(1-z)}{z}-\frac{\log z}{1-z}\right)d\;z\\ =\frac{\log(1-x)\cdot \log x}{y}-\left(\frac{1}{yx^{y}}+\frac{\log x}{x^{y}}\right)\int_{0}^{x}\log\left(1-z\right)\cdot z^{y-1}d\;z+\frac{1}{yx^{y}}\int_{0}^{x}\frac{z^{y}\log z}{1-z}d\;z\\ =-\frac{\log(1-x)}{y^{2}}-\left(\frac{1}{y^{2}}+\frac{\log x}{y}\right)g_{1}\left(x,y\right)+\frac{g_{2}\left(x,y\right)}{y}. \end{array}$$

Therefore, the average VoI is derived by substituting (A8) and (A9) into (A7).

The lower bound in (25) is obtained by applying Jensen’s inequality, i.e., the following:

(A10) $$\begin{array}{cc} E[V] & =E\left[-\frac{1}{2}\log\left(1-\frac{\gamma }{1+\gamma }e^{-2\kappa a}\right)\right]\\ \; & \geq -\frac{1}{2}\log\left(1-\frac{\gamma }{1+\gamma }E\left[e^{-2\kappa a}\right]\right), \end{array}$$

where

(A11) $$\begin{array}{cc} E[e^{-2\kappa a}] & =\int_{0}^{+\infty }e^{-2\kappa a}f_{A}\left(a\right)d\;a\\ \; & =\frac{\frac{\mu -\lambda }{2\kappa }}{\frac{\mu -\lambda }{2\kappa }+1}-\frac{\frac{\mu -\lambda }{2\kappa }\left(\frac{\mu +\lambda }{2\kappa }+1\right)}{{\left(\frac{\mu }{2\kappa }+1\right)}^{2}\left(\frac{\lambda }{2\kappa }+1\right)}. \end{array}$$

The upper bound in (26) is the average VoI in the Markov OU process. In the hidden Markov model, we can write the following [31]:

(A12) $$\begin{array}{cc} v(t) & =h\left(X_{t}\right)-h\left(X_{t}|Y_{t{}_{n}^{\prime }},\ldots ,Y_{t{}_{n-m+1}^{\prime }}\right)\\ \; & \leq h\left(X_{t}\right)-h\left(X_{t}|Y_{t{}_{n}^{\prime }},\ldots ,Y_{t{}_{n-m+1}^{\prime }},X_{t_{n}}\right)\\ \; & =h\left(X_{t}\right)-h\left(X_{t}|X_{t_{n}}\right)\\ \; & =I(X_{t};X_{t_{n}}). \end{array}$$

Therefore, the VoI in the Markov model can be regarded as the upper bound of the VoI in the hidden Markov model. Denote $v_{\mathrm{OU}}\left(t\right)=I\left(X_{t};X_{t_{n}}\right)$ as the VoI in the underlying OU process. Then, the result in (26) follows from the following calculation:

(A13) $$\begin{array}{c} E\left[V\right]\leq E\left[V_{\mathrm{OU}}\right]=E\left[-\frac{1}{2}\log\left(1-e^{-2\kappa a}\right)\right]\\ =-\frac{\mu }{4\kappa }\int_{0}^{1}\log(1-r)\left[\frac{\mu -\lambda }{\mu }r^{\frac{\mu -\lambda }{2\kappa }-1}+\frac{\lambda }{\mu -\lambda }r^{\frac{\lambda }{2\kappa }-1}-\left(\frac{\mu }{\mu -\lambda }-\frac{\lambda }{\mu }-\frac{\lambda }{2\kappa }\log r\right)r^{\frac{\mu }{2\kappa }-1}\right]d\;r. \end{array}$$

Similar to the calculation given in (A8) and (A9), for the given *y*, we have the following:

(A14) $$\begin{array}{cc} \int_{0}^{1}\log\left(1-r\right)\cdot r^{y-1}d\;r & =-\frac{1}{y}H\left(y\right),\\ \int_{0}^{1}\log r\cdot \log\left(1-r\right)\cdot r^{y-1}d\;r & =\frac{1}{y^{2}}H\left(y\right)-\frac{1}{y}\varphi^{\left(1\right)}\left(y\right). \end{array}$$

Therefore, the upper bound of the average VoI is derived by substituting (A14) into (A13).

## Appendix C. Proof of Proposition 4

The MGF of the VoI is obtained directly by the following:

(A15) $$\begin{array}{c} M_{v}\left(t\right)=\int_{0}^{\frac{1}{2}\log\left(1+\gamma \right)}e^{tv}f_{V}\left(v\right)d\;v=\frac{\mu }{2\kappa }\int_{0}^{1}{\left(1-\frac{\gamma }{1+\gamma }r\right)}^{-\frac{t}{2}}[\frac{\mu -\lambda }{\mu }r^{\frac{\mu -\lambda }{2\kappa }-1}\\ +\frac{\lambda }{\mu -\lambda }r^{\frac{\lambda }{2\kappa }-1}-\left(\frac{\mu }{\mu -\lambda }-\frac{\lambda }{\mu }-\frac{\lambda }{2\kappa }\log r\right)r^{\frac{\mu }{2\kappa }-1}]d\;r. \end{array}$$

Here, for the given *x*, *y* and *t*, we have the following [30]:

(A16) $$\int_{0}^{1}{\left(1-xr\right)}^{-\frac{t}{2}}\cdot r^{y-1}d\;r=\frac{1}{y}{}_{2}F_{1}\left(y,\frac{t}{2};y+1;x\right),$$

and

(A17) $$\int_{0}^{1}\log r\cdot {\left(1-xr\right)}^{-\frac{t}{2}}\cdot r^{y-1}d\;r=-\frac{1}{y^{2}}{}_{3}F_{2}\left(y,y,\frac{t}{2};y+1,y+1;x\right).$$

Therefore, the MGF of VoI is derived by substituting (A16) and (A17) into (A15).
